# Do pigs have altruistic behaviour based on cognitive empathy?

**DOI:** 10.1186/s12917-026-05495-8

**Published:** 2026-04-18

**Authors:** Yaqian Zhang, Jiaqi Yu, Fang Sun, Yuhan Yao, Wenqi Li, Ziyu Bai, Xiangyu Liu, Xiang Li

**Affiliations:** 1 College of Animal Science and Technology, Northeast Agricultural University, Harbin, China; 2 Office of Laboratory Management, Northeast Agricultural University, Harbin, China; 3 College of Life Science, Northeast Agricultural University, Harbin, China

**Keywords:** Piglet, Altruistic behaviour, Cognitive empathy, Emotion, Welfare

## Abstract

**Background:**

Altruistic behaviour is commonly defined as actions that benefit others at a potential cost to oneself and, in animal research, is typically inferred when individuals assist conspecifics without immediate personal gain. In pigs, altruistic behaviour associated with cognitive empathy remains an understudied area. In the present study, altruistic behaviour was operationally defined as a pig voluntarily sacrificing its own access to a food reward to alleviate a peer’s distress. We investigated whether pigs adjust their own behaviour in relation to the emotional state of others in a manner consistent with cognitive empathy-based altruism.

**Methods:**

A total of 180 piglets were assigned to 90 test units, which were randomly allocated to one of nine treatment groups in a 3 × 3 factorial design. In this design, both the active and passive pigs could receive one of three treatments (reward: food, punishment: mild electric shock, or no treatment) after the active pig pressed the button. Behavioural manifestations and changes in heart rate of both active and passive pigs were recorded for one hour.

**Results:**

The results showed that when passive pig was punished, active pig reduced the button presses to alleviate passive pig’s pain, even though active pig itself would receive a reward (*p <* 0.01).

**Conclusion:**

This behaviour suggests that pigs are able to relate their own behaviour to the emotional distress of their peers and act to alleviate the distress, demonstrating altruistic behaviour driven by cognitive empathy.

## Background

Some animals exhibit altruistic behaviour, defined as responding to another individual’s need by providing assistance, even when doing so yields no immediate direct benefit to the helper [1]. Such behaviour has been documented not only among familiar conspecifics but also towards unrelated or unfamiliar individuals, suggesting that altruistic responses may extend beyond close social partners [1]. Such behaviour may include helping, protection, food sharing, or refraining from actions that could harm another individual, which has been reported in rodents, non-human primates, and other social mammals [2–5]. From an evolutionary perspective, these behaviours may contribute to social cohesion, reciprocal interactions, or inclusive fitness, thereby promoting group stability [6]. Empathy describes the ability to feel and understand the emotions of others and is a fundamental element of social interaction weaving together cognitive and emotional recognition, regarded as an essentially mammalian heritage [7]. The concept of empathy encompasses not only emotional contagion but also cognitive empathy [8]. Empathy is widely regarded as a proximate motivational mechanism underlying prosocial and altruistic behaviours, as the perception of another’s distress can elicit affective responses that promote supportive or helping actions [9, 10]. Unlike simple behavioural synchrony, empathic processes imply that the observer’s emotional state is influenced by, and linked to, the emotional experience of another individual.

The conceptual relationship between empathy and altruism has been discussed extensively in both psychological and evolutionary frameworks. De Waal (2008) introduced the concept of “directed altruism”, referring to helping behaviour specifically triggered by another individual’s pain, need, or distress [11]. Within this framework, empathy functions as an underlying mechanism that transforms the perception of another’s negative emotional state into a motivation to act. De Waal and Preston’s Russian doll model to explain empathy, where simpler emotional processes are at the core, and increasing cognitive abilities leading to more sophisticated forms of empathy [1]. Through such mechanisms, empathy is thought to facilitate coordinated behaviour, reduce social tension, and enhance group functioning [12].

Previous studies have demonstrated that pigs are capable of empathy and may be influenced by the emotional states of their conspecifics, showing evidence of emotional contagion when observing group members in stressful situations [13]. For example, Reimert et al. reported that pigs exposed to conspecifics experiencing aversive events exhibited increased stress-related behaviours, such as ears held back and alert standing [14]. This suggests that the inexperienced pigs perceived the negativity of demonstrator pigs, which triggered their own negative emotions [15]. The above studies have demonstrated that emotional contagion can occur in pigs. In our previous studies, we found that pigs were influenced by the emotional states of their conspecifics regardless of whether they understood the source of those emotions. They exhibited prosocial motivation and empathic-like responses, providing social support to their companions [16, 17]. In particular, increased interaction with conspecifics in positive emotional states was associated with improved cognitive performance, reduced anxiety-like behaviours, and enhanced environmental adaptability [18]. Building on these findings, we proposed that piglets may exhibit behavioural patterns indicative of altruistic tendencies grounded in cognitive empathy. However, due to methodological limitations in previous studies, this hypothesis has not yet been directly tested.

Individuals producing altruistic behaviour appear to be motivated by cognitive empathy resulting from the needs of others [19]. The objective of this study was to investigate the relationship between cognitive empathy and altruistic behaviour in pigs. Since pigs are able to recognise the emotions of their peers and are able to proactively provide social support to their peers who are in a negative emotional state, we hypothesise that pigs are not only able to recognise the emotions of their peers, but are also able to determine the source of their peers’ emotions and take action to help their peers to alleviate their negative emotions, i.e., pigs have the presence of altruistic behaviours based on cognitive empathy. If a pig displays altruistic behaviour, it is an indication that the pig has the ability to alleviate the suffering of its companion. If the cognitive empathy of pigs is instinct, it could provide a novel pathway into the alleviation of negative emotions within the pigs and the improvement of the welfare of pigs in production.

## Methods

### Ethics

The experiment was conducted in accordance with Chinese laws and regulations and was approved by the Laboratory Animal Ethics Committee of Northeast Agricultural University. The study was conducted under accreditation NEAUEC2021 02 14 and was regulated throughout by the Laboratory Animal Ethics Committee of Northeast Agricultural University. Piglet manipulation during both the test and before test was within limits of regular husbandry procedures. After the experiment, all the piglets returned to the production process.

### Animals

A total of 180 piglets (purebred Large White piglets) were selected from 30 litters (six piglets randomly selected from each litter). All piglets originated from routine matings for commercial piglet production at the Animal Farm of Northeast Agricultural University in Shuangcheng District, Harbin, Heilongjiang Province. No targeted matings were made for the study. All male piglets in the selected litters underwent a procedure to castration them at seven days of age, after which they were uniquely ear-tagged. This experiment was conducted from March to April at the Animal Farm of Northeast Agricultural University in Shuangcheng District, Harbin, Heilongjiang Province. The age difference among all 30 litters did not exceed 14 days. At weaning (Mean ± SD: 29.00 ± 8.06 days of age), six piglets were randomly selected from each litter. The remaining littermates were removed from the pen following selection. The selected piglets were housed together by litter in a single metal pen (3.60 m×2.50 m×1.20 m, 1.5 m²/pig), with a total of 30 pens located within the same barn. From weaning until the completion of testing at 71 days of age, the six piglets from each litter remained housed together in the same pen and were not mixed with unfamiliar individuals. Consequently, social relationships within each test group remained stable throughout the rearing period and during the experimental procedures. The pens were covered with rice husk of 0.50 m thickness as bedding without any supplement or replacement during the experiment. The pig house was naturally lit and the windows were opened for natural ventilation from 10:00 to 14:00 daily. The temperature was 20.00–25.00 ℃ (23.02 ± 1.92 ◦C) and the air humidity was kept at 65.00–75.00% (70.12 ± 2.96%).

All piglets had access to water ad libitum, and were fed three times per day, at 7:00, 12:00, and 16:00, according to 50 g/kg/head feeding (energy of 3.30 MJ/kg, crude protein 17.00%, crude fiber 4.00%, lysine 1.30%). All operators were required to wear the same overalls throughout the experiment. Stress treatment was avoided during the feeding procedures and routine immunization procedures were carried out.

### Experimental set-up

**Fig. 1:**
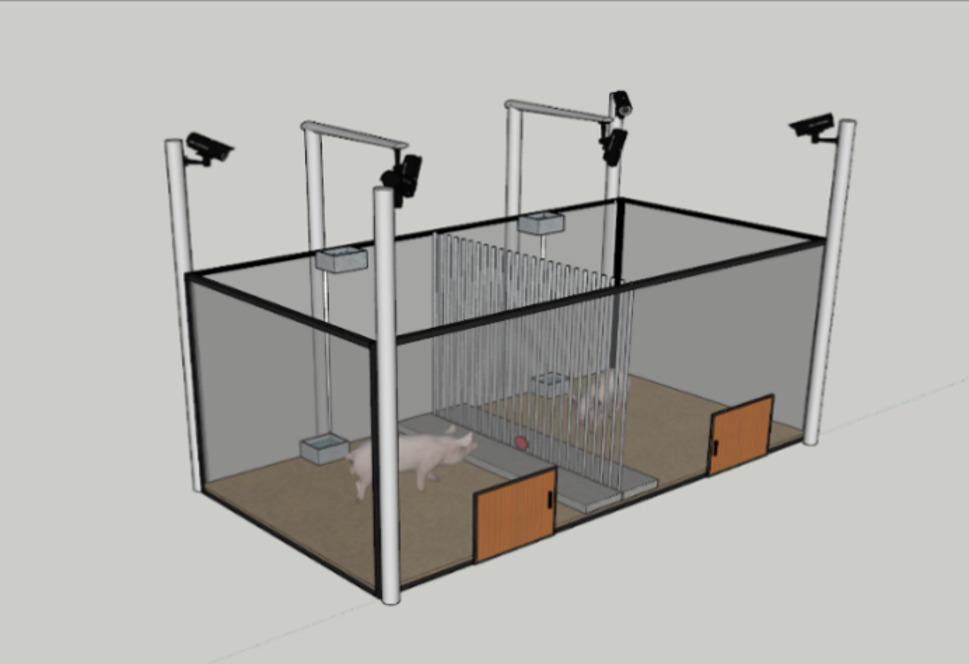
Three-dimensional structure of the test box

This test uses an empathic response test box (China Patent, ZL 202211498851.4, as shown in Fig. 1), and a button is fixed in the middle of the fence on the active pig side, in such a way that the pig pressing down the button could easily press it, and the passive pig could not directly observe the button. The button was mounted vertically on the active pig’s side of the partition and oriented towards the active pig. An opaque backing plate was attached to the rear of the button, facing the passive pig compartment, in order to reduce the passive pig’s visual attention to the active pig’s button pressing. This configuration allowed the passive pig to perceive the outcomes of the active pig’s actions (i.e., the aversive stimulus and the active pig’s behavioural responses) whilst preventing direct visual observation of button manipulation. This arrangement minimised the likelihood that changes in active pig behaviour were driven by simple visual cues from the passive pig observing button manipulation. Eighteen test boxes were used for the test. All measurements and spatial arrangements were consistent across all test units to minimise apparatus-related variability.

The test box is a rectangular shape (300 cm × 150 cm × 130 cm) made of grey acrylic sheets, polyurethane foam and wood panels with an uncovered top. The test box was separated in the middle by a metal railing (railing diameter 0.5 cm, length 130 cm), with a spacing of 5 cm between adjacent rails, separating the test box into two axisymmetric square test areas (side length 150 cm), placing a platform with a height of 5 cm on each side of the railing (150 cm × 25 cm × 5 cm), and covering all other areas with 5 cm thick rice husk. The automatic water dispenser and sink (20 cm × 17 cm × 10 cm) fixed to the wall were parallel to the partition and 55 cm and 65 cm away from the railing, respectively. Doors (40 cm × 50 cm) were installed on the opposite wall of the troughs, and a total of six surveillance cameras were mounted on the four corners and two tops of the test box, 200 cm above the floor, to record pig behaviour.

### Test group

Prior to testing, two piglets of similar body weight from the same litter were paired to form a test unit, yielding a total of 90 test units (180 piglets), which were then randomly allocated to 9 groups with 10 replicates each. Piglets of both sexes were randomly assigned to test units and treatment groups. Within each test unit, the roles of active pig and passive pig were assigned at random prior to testing, so that no systematic bias related to body weight, sex, or individual behavioural characteristics influenced role allocation. Each test unit (one active pig - passive pig dyad) was exposed to only one treatment condition. When active pig pressed the button, active pig and passive pig received three different treatments. The specific treatment conditions for each group are presented in Table 1. The three treatments include: (1) reward treatment: a food reward (10 g of feed + 5 g of apple) was offered to the subject piglets under fasting conditions and placed in the trough of the test box; (2) punishment treatment: an electric shock prod (for 0.2 s) to the rump of the subject piglets with a commercially available pig catching stick (Musyder, Guangzhou, Output voltage 7.4 ~ 8.4 V, output current 0.7 A); (3) no treatment: the active pig presses the button without any treatment, leaving the piglets in their original condition. In each group, a total of 10 replicate pairs were available, from which six replicates were randomly selected for behavioural observations, and the remaining four replicates were used for heart rate recording.

**Table 1:** Experimental treatment groups

Test Group	Active Pig Treatment	Passive Pig Treatment
ART-PRT	Reward	Reward
APT-PRT	Punishment	Reward
ANT-PRT	No Treatment	Reward
ART-PPT	Reward	Punishment
APT-PPT	Punishment	Punishment
ANT-PPT	No Treatment	Punishment
ART-PNT	Reward	No Treatment
APT-PNT	Punishment	No Treatment
ANT-PNT	No Treatment	No Treatment

*ART* Reward treatment of active pig, *APT* Punishment treatment of active pig, *ANT* No treatment of active pig, *PRT* Reward treatment of passive pig, *PPT* Punishment treatment of passive pig, *PNT* No treatment of passive pig

### Habituation

All piglets were familiarized with the presence and proximity of the experimenter during routine daily feeding and husbandry procedures for at least two weeks prior to testing. At 70 days of age, the piglets were familiarized with the test box environment. The test box was located in a quiet room adjacent to the home pens. Each test unit was gently transferred from the home pen to the test box via a covered corridor. To ensure that the buttons were a novelty to the piglets, no buttons were placed in the test box during familiarization. At 8:00 a.m., each group of piglets was guided to two areas in the test box, separated by a metal partition according to the grouping of each test unit. In these areas, they could drink freely but not feed, and the piglets were allowed to move freely. The time allowed for the piglets to familiarize themselves with the environment was one hour. During the habituation period, the experimenter stayed in the room, outside the test arena, hidden from the piglets’ view. After the habituation period, the piglets were returned to their original pens. Piglets that reached the same target age on the same experimental day were habituated simultaneously in different test boxes. After the habituation period for each unit, the test box was thoroughly cleaned and allowed to dry completely.

### Tests

Different treatment groups were tested in parallel within the same time window in different test boxes. Piglets reaching the same age were tested on the same experimental day, with a maximum of nine test units conducted per day. The piglets were not fed at 7:00 a.m. on the day of the test. At 08:00 a.m., the active pig and passive pig were transferred to the test box by the experimenter. Between 08:00 a.m. and 08:20 a.m., the pigs remained in the box for habituation; during this period the button was covered and could not be operated. The formal test started at 8:20 a.m., after which the pigs were allowed to explore freely for 1 h, during which time the active pig could press the button at any time. This duration was determined based on our previous research on emotional contagion [18]. Button was positioned on the active pig’s side in the test box, whereas no button was accessible to the passive pig. During the experimental procedure, each time the active pig pressed a button, the experimenter applied a corresponding treatment to the active pig and passive pig. When administering the treatment, the passive pig received the treatment first, immediately followed by the active pig, and all experimenters were hidden from the pigs’ view. Once the test was complete, the pigs were returned to their original pens. To prevent the transfer of information between the returning pigs and those remaining in the test units, selected test units from the same litter completed the test simultaneously in different test boxes. To prevent potential carry-over effects, the test box was thoroughly cleaned after each test.

### Behavioural analyses

Piglets’ behaviour was continuously video-recorded for 60 min during the test period (08:20–09:20) using six digital video cameras (Hangzhou Hikvision Digital Technology Co. Ltd., Hangzhou, China) positioned at different orientations above the test box. Behavioural analysis was conducted using Focal Sampling with Continuous Recording for all pigs. The following behaviour was analysed: the frequency (N, occurrences in the one observing hour) of nose-to-nose contact, nose-to-partition contact, freezing, escape attempts, proximity, and button presses (button presses only for active pigs), as well as the duration (min, in the one observing hour) of exploring. Specific behavioural definitions are provided in Table 2 [13, 14, 20]. And to reduce errors due to differences in recording personnel, all behavioural recordings were completed by the same experimenter.

**Table 2:** Behavioural parameters and definitions

Parameter	Description
Button presses(N)	The pig pressed the button with its snout
Nose-to-nose contacts(N)	The initiator pig touches the snout of a penmate with its snout (< 2 s)
Nose-partition contact(N)	The pig touches the metal fence in the middle with its snout
Proximity(N)	Frequency of times a pig’s head enters an area less than or equal to 25 cm from the fence
Escape attempts(N)	Pigs jumping towards the door or middle fence of the test box
Freezing(N)	Standing motionless with whole body and head fixed
Exploring(min)	Nose sniffing and arching around rice hulls and test boxes in the rice hull area

### Heart rate analysis

Heart rate monitors (Lifeon ECG Recorder, DitPatch-H1, Shenzhen, China) were fitted at 08:00 a.m. on the test day (71 days of age). The monitor consisted of a flexible thoracic belt with two integrated electrodes and a wireless data transmission unit. Electrode positions were adjusted until stable heart rate signals were obtained. The subsequent 20-minute period (08:00–08:20 a.m.) served as a habituation phase, allowing piglets to acclimatise to the monitor and the test box. Following the method described by Zhang et al. [17], the mean heart rate recorded during 08:11–08:20 a.m. was defined as the resting baseline value for each piglet. The formal test commenced at 08:20 a.m. and lasted for 1 h. Heart rate was continuously monitored throughout the test, and mean values were calculated for each 5-minute interval, yielding 12 average heart rate values per piglet. Heart rate data were expressed as change relative to the resting heart rate (*X*), calculated as follows:

$$\:\mathrm{X}{(\%)\:=}\frac{{\mathrm{y}}_{\text{mean heart rate}}\:\mathrm{-}{\mathrm{y}}_{\text{resting heart rate}}}{{\mathrm{y}}_{\text{resting heart rate}}}\times{100}{\%}$$

### Statistical analysis

Data were statistically analyzed by SPSS 21 (SPSS Inc., Chicago, IL, USA). All data were examined for normal distribution using Kolmogorov–Smirnov test. All data were tested to be normally distributed. The effects of different treatments of active pig or passive pig on pigs’ behaviour were analyzed using two-way ANOVA. All behavioural parameters were significantly affected by the interaction between active pig treatment and passive pig treatment (all *p* ≤ 0.05). The statistical model of the two-way ANOVA was as follows: *Yij = µ + Ai + Bj + (AB)ij + e*; *Yij* is the target trait, *µ* is the overall mean, *Ai* is the different treatment of active pig (reward, punishment, or nothing, 3 levels), *Bj* is the different treatment of passive pig (reward, punishment, or nothing, 3 levels), *(AB)ij* is the interaction, and *e* is the random error. One-way ANOVA was performed on the data within the different test groups for active pig and passive pig. The statistical model of the one-way ANOVA was as follows: *Yi = µ + Ai + e*; *Yi* is the target trait, *µ* is the overall mean, *Ai* is the different treatment (reward, punishment, or nothing, 3 levels), and *e* is the random error. Heart rate data were analysed using the same approach as for behavioural data, and the temporal profile of the mean heart rate change rate over one hour is presented as a curve to illustrate the overall trend. Post-hoc multiple comparisons were conducted using Tukey’s honestly significant difference (HSD) test. Effects and differences were considered significant if *p* ≤ 0.05.

## Results

### The button-press frequency of the active pigs

**Fig. 2:**
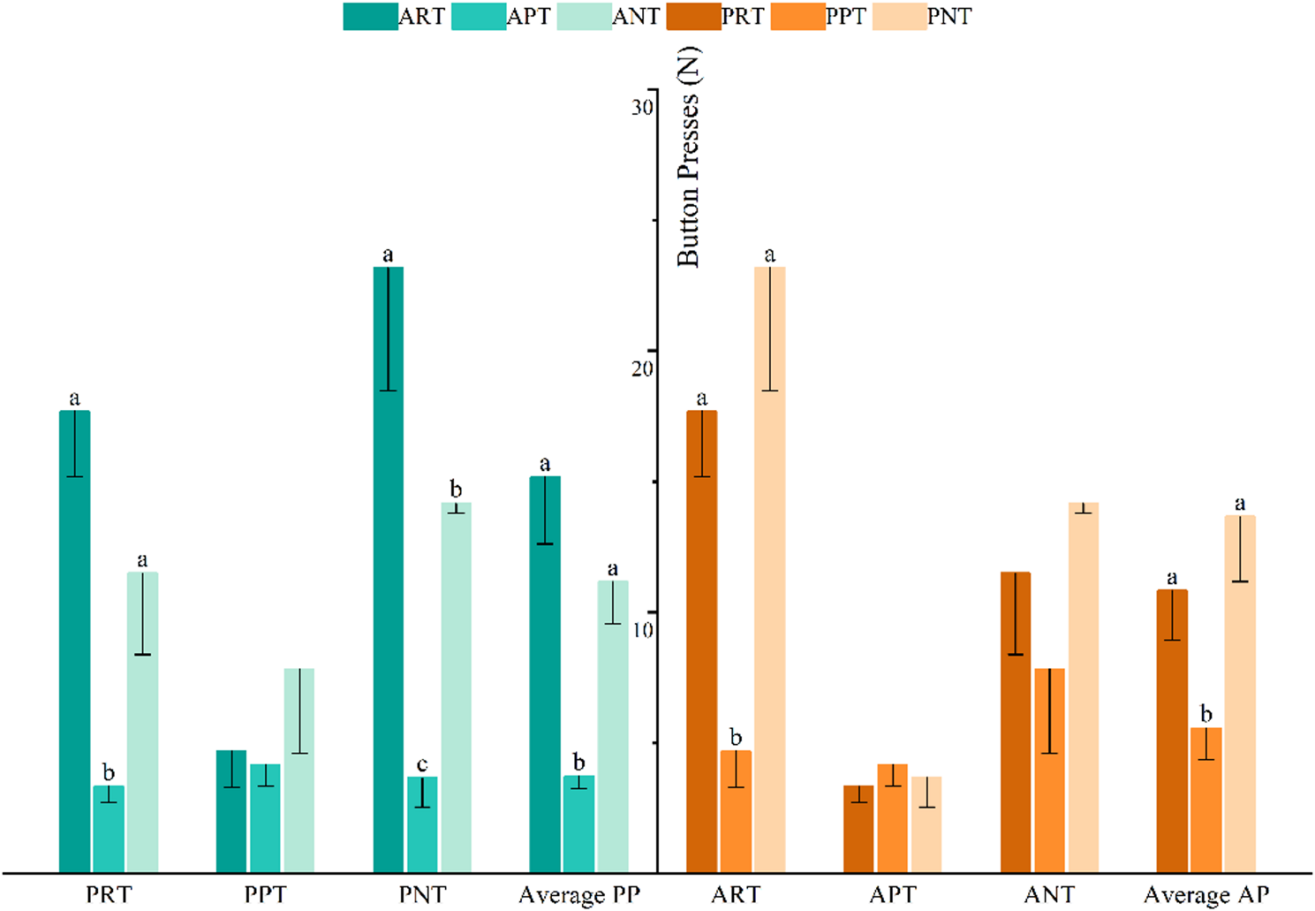
Effect of different treatments of active pig and passive pig on the frequency of active pig button presses. ART: Reward treatment of active pig; APT: Punishment treatment of active pig; ANT: No treatment of active pig; PRT: Reward treatment of passive pig; PPT: Punishment treatment of passive pig; PNT: No treatment of passive pig. The left side of the horizontal axis represents the different treatments of the passive pig, while the right side represents the different treatments of the active pig. The vertical axis represents the frequency of button presses by the active pig. Different letters denote significant differences (*n* = 6, *p* ≤ 0.05)

Figure 2 shows the effects of treatments applied to the active and passive pigs on the button-pressing behaviour by the active one. Results are presented as Means ± SE. Different treatments of active pig have a significant effect on the button presses. Punishment treatment (3.72 ± 0.48) results in the lowest average button presses for active pigs compared to the reward treatment (15.17 ± 2.55) and no treatment (11.17 ± 1.60, *p* < 0.01). Within passive pig reward treatment group, the button presses are significantly greater in both the reward treatment (17.67 ± 2.47) and no treatment of active pig (11.50 ± 3.12) than in the punishment treatment (3.33 ± 0.61, *p* < 0.01). Within passive pig no treatment group, all treatments of active pig differ significantly from each other on the button presses, with punishment treatment (3.67 ± 1.12) causing the lowest button presses and reward treatment the highest (23.17 ± 4.71, *p* < 0.01). Different treatments of passive pigs have a significant effect on the button presses. Punishment treatment results in the lowest average button presses (5.56 ± 1.20) compared to the reward treatment (10.83 ± 1.90) and no treatment (13.67 ± 2.49, *p* < 0.01). Within active pig reward treatment group, the button presses are significantly greater in both the reward (17.67 ± 2.47) and no treatment of passive pig (23.17 ± 4.71) than in the punishment treatment (4.67 ± 1.36, *p* < 0.01). There are no significant differences between the different treatments in the remaining groups (*p* > 0.05).

### Behaviour of active pigs

Table 3 shows the effect of different treatments of active and passive pigs on the behaviour of active one. Different treatments of active pig have an impact on the behaviour of active pig. All treatments differ significantly from each other on the proximity, with punishment treatment causing the lowest proximity and active pig reward treatment the highest (*p* < 0.01). Punishment treatment results in the highest average nose-to-nose contacts (*p* < 0.01), average freezing (*p* < 0.01), and average escape attempts (*p* < 0.01) compared to the other treatments. Within passive pig reward group, the punishment treatment results in the highest freezing (*p* < 0.01) and escape attempts (*p* < 0.01), while results in the lowest proximity (*p* < 0.01) compared to the others. Within passive pig punishment group, the punishment treatment of active pig results in the highest nose-to-nose contacts (*p* = 0.04) and escape attempts (*p* < 0.01) compared to the others, and the freezing is significantly greater than in the no treatment (*p* < 0.01). Within passive pig no treatment group, the reward treatment results in the highest proximity (*p* < 0.01) and in the lowest freezing (*p* = 0.01) compared to the other treatments, while punishment treatment results in the highest escape attempts compared to the others (*p* < 0.01). Different treatments of passive pig have an impact on the behaviour of active pig. The punished treatment results in the highest average freezing compared to the other treatments of passive pig (*p* < 0.01). No treatment causes the highest average proximity compared to the others (*p* = 0.02). Within active pig reward group, the punishment treatment results in the highest freezing compared to the other treatments (*p* < 0.01), while no treatment causes the highest proximity compared to the others (*p* = 0.01). There are no significant differences between the different treatments in the remaining groups (*p* > 0.05).

**Table 3:** Behaviour of active pigs after different treatments of active pig and passive pig (*n* = 6)

		PRT	PPT	PNT	Average	*p*
Nose-to-nose contacts	ART	3.17 ± 0.87	2.67 ± 0.67^B^	2.17 ± 0.48	2.67 ± 0.39^B^	0.60
	APT	6.50 ± 1.48	7.17 ± 2.02^A^	5.67 ± 1.48	6.44 ± 0.92^A^	0.82
	ANT	3.33 ± 0.84	2.67 ± 0.49^B^	4.83 ± 1.14	3.61 ± 0.52^B^	0.23
	Average	4.33 ± 0.71	4.17 ± 0.86	4.22 ± 0.70		0.98
	*p*	0.09	0.04*	0.10	< 0.01*	
Nose-partition contacts	ART	6.33 ± 1.31	6.67 ± 1.56	4.83 ± 0.79	5.94 ± 0.71	0.56
	APT	8.00 ± 1.81	6.00 ± 1.57	3.17 ± 1.08	5.72 ± 0.95	0.11
	ANT	4.83 ± 1.35	5.00 ± 0.82	6.50 ± 1.23	5.44 ± 0.65	0.55
	Average	6.39 ± 0.88	5.89 ± 0.76	4.83 ± 0.66		0.35
	*p*	0.36	0.69	0.11	0.90	
Proximity	ART	22.00 ± 3.15^Aab^	16.17 ± 1.74^b^	28.00 ± 1.79^Aa^	22.06 ± 1.72^A^	0.01*
	APT	8.33 ± 1.23^B^	12.33 ± 2.25	11.00 ± 2.96^B^	10.56 ± 1.29^C^	0.46
	ANT	16.00 ± 1.57^A^	11.00 ± 1.55	15.83 ± 2.75^B^	14.28 ± 1.24^B^	0.18
	Average	15.44 ± 1.80^b^	13.17 ± 1.14^b^	18.28 ± 2.22^a^		0.02*
	*p*	< 0.01*	0.16	< 0.01*	< 0.01*	
Freezing	ART	8.83 ± 2.04^Bb^	39.17 ± 3.91^aAB^	11.00 ± 1.88^Bb^	19.67 ± 3.67^B^	< 0.01*
	APT	42.83 ± 9.99^A^	56.50 ± 3.53^A^	35.83 ± 6.96^A^	45.06 ± 19.01^A^	0.16
	ANT	8.83 ± 1.64^B^	22.00 ± 9.22^B^	28.83 ± 4.96^A^	19.89 ± 3.88^B^	0.10
	Average	20.17 ± 5.06^b^	39.22 ± 4.77^a^	25.22 ± 3.73^b^		< 0.01*
	*p*	< 0.01*	< 0.01*	0.01*	< 0.01*	
Escape attempts	ART	1.17 ± 0.48^B^	1.83 ± 0.65^B^	1.83 ± 0.65^B^	1.61 ± 0.34^B^	0.67
	APT	7.00 ± 1.00^A^	8.67 ± 1.54^A^	6.33 ± 1.20^A^	7.33 ± 0.73^A^	0.43
	ANT	1.33 ± 0.49^B^	2.17 ± 0.87^B^	0.67 ± 0.33^B^	1.39 ± 0.36^B^	0.25
	Average	3.17 ± 0.76	4.22 ± 0.97	2.94 ± 0.74		0.18
	*p*	< 0.01*	< 0.01*	< 0.01*	< 0.01*	
Exploring	ART	7.91 ± 2.73	5.11 ± 1.76	4.15 ± 2.13	5.73 ± 1.28	0.49
	APT	2.92 ± 0.73	5.56 ± 1.86	4.60 ± 2.79	4.36 ± 1.11	0.65
	ANT	11.18 ± 4.63	5.12 ± 2.27	3.34 ± 0.98	6.55 ± 1.83	0.19
	Average	7.34 ± 1.89	5.26 ± 1.07	4.03 ± 1.15		0.26
	*p*	0.21	0.98	0.91	0.55	

*ART* Reward treatment of active pig, *APT* Punishment treatment of active pig, *ANT* No treatment of active pig, *PRT* Reward treatment of passive pig, *PPT* Punishment treatment of passive pig, *PNT* No treatment of passive pig. Mean ± standard error. Different letters denote significant differences (a/b/c: different treatments of passive pigs; A/B/C: different treatments of active pigs)

### Behaviour of passive pigs

Table 4 shows the effect of different treatments of active pig and passive pig on passive pig’s behaviour. Different treatments of active pig have an impact on the behaviour of passive pig. The punishment treatment results in a significant increase in average nose-to-nose contact over the reward treatment (*p* = 0.02). No treatment results in the lowest average exploring compared to the other treatments (*p* = 0.03). Within passive pig reward group, the punishment treatment of active pig results in the highest average freezing compared to the others (*p* < 0.01), while causes a significant increase in average proximity over the no treatment (*p* = 0.02). Within passive pig punishment group, no treatment results in the highest escape attempts compared to the others (*p* < 0.01). Different treatments of passive pig have an impact on the behaviour of passive pig. The punishment treatment results in the highest average nose-to-nose contacts (*p* < 0.01), average nose-partition contacts (*p* = 0.02), average freezing (*p* < 0.01), and average escape attempts (*p* < 0.01) compared to the other treatments. Within active pig reward group, the punishment treatment of passive pig results in the highest freezing (*p* < 0.01) and escape attempts (*p* < 0.01) compared to the other treatments. Within active pig punishment group, the punishment treatment of passive pig results in the highest escape attempts compared to the other treatments (*p* < 0.01). Within active pig no treatment group, the punishment treatment results in the highest nose-to-nose contact (*p* = 0.05), freezing (*p* < 0.01), and escape attempts (*p* < 0.01) compared to the other treatments. There are no significant differences between the different treatments in the remaining groups (*p* > 0.05).

**Table 4:** Behaviour of passive pigs after different treatments of active pig and passive pig (*n* = 6)

		PRT	PPT	PNT	Average	*p*
Nose-to-nose contacts	ART	2.50 ± 0.56	3.33 ± 0.49	2.33 ± 0.62	2.72 ± 0.32^B^	0.42
	APT	3.83 ± 0.79	7.67 ± 1.78	4.50 ± 1.09	5.33 ± 0.81^A^	0.11
	ANT	3.00 ± 0.68^b^	6.00 ± 1.32^a^	2.67 ± 0.67^b^	3.89 ± 0.63^AB^	0.05*
	Average	3.11 ± 0.40^b^	5.67 ± 0.83^a^	3.17 ± 0.50^b^		< 0.01*
	*p*	0.40	0.09	0.17	0.02*	
Nose-partition contacts	ART	4.00 ± 0.68	4.17 ± 0.87	4.00 ± 1.53	4.06 ± 0.59	0.99
	APT	3.33 ± 0.80	13.67 ± 4.54	4.33 ± 2.55	7.11 ± 2.00	0.06
	ANT	3.67 ± 0.99	6.83 ± 1.54	3.67 ± 0.67	4.72 ± 0.71	0.10
	Average	3.67 ± 0.46^b^	8.22 ± 1.81^a^	4.00 ± 0.96^b^		0.02*
	*p*	0.85	0.08	0.97	0.21	
Proximity	ART	21.50 ± 1.88^AB^	15.83 ± 3.00	15.83 ± 1.78	17.72 ± 1.40	0.16
	APT	27.83 ± 2.52^A^	21.33 ± 6.33	16.67 ± 2.94	21.94 ± 2.57	0.21
	ANT	15.83 ± 3.48^B^	15.50 ± 3.21	17.67 ± 4.11	16.33 ± 1.98	0.90
	Average	21.72 ± 1.89	17.56 ± 2.50	16.72 ± 1.69		0.18
	*p*	0.02*	0.59	0.92	0.14	
Freezing	ART	6.50 ± 2.14^Bb^	50.33 ± 7.50^a^	11.67 ± 2.85^b^	22.83 ± 5.41	< 0.01*
	APT	33.17 ± 3.93^A^	45.83 ± 7.04	24.17 ± 6.86	34.39 ± 3.95	0.07
	ANT	7.33 ± 2.06B^c^	61.50 ± 5.07^a^	24.17 ± 5.52^b^	31.00 ± 6.01	< 0.01*
	Average	15.67 ± 3.38^b^	52.56 ± 3.93^a^	20.00 ± 3.23^b^		< 0.01*
	*p*	< 0.01*	0.26	0.20	0.28	
Escape attempts	ART	1.50 ± 0.67^b^	8.00 ± 0.82^aB^	2.00 ± 0.68^b^	3.83 ± 0.82	< 0.01*
	APT	2.67 ± 0.67^b^	9.83 ± 0.83^aB^	2.50 ± 0.95^b^	5.00 ± 0.94	< 0.01*
	ANT	1.00 ± 0.37^b^	14.00 ± 0.73^Aa^	2.00 ± 0.58^b^	5.67 ± 1.47	< 0.01*
	Average	1.72 ± 0.36^b^	10.61 ± 3.16^a^	2.17 ± 0.41^b^		< 0.01*
	*p*	0.15	< 0.01*	0.87	0.50	
Exploring	ART	8.92 ± 3.14	8.06 ± 2.34	9.13 ± 3.52	8.70 ± 1.65^A^	0.97
	APT	7.90 ± 2.11	7.56 ± 2.37	11.34 ± 3.09	8.93 ± 1.45^A^	0.53
	ANT	5.73 ± 1.66	3.57 ± 0.79	3.86 ± 1.33	4.39 ± 0.75^B^	0.47
	Average	7.52 ± 1.33	6.40 ± 1.18	8.11 ± 1.70		0.69
	*p*	0.64	0.24	0.19	0.03*	

*ART* Reward treatment of active pig, *APT* Punishment treatment of active pig, *ANT* No treatment of active pig, *PRT* Reward treatment of passive pig, *PPT* Punishment treatment of passive pig, *PNT* No treatment of passive pig. Mean ± standard error. Different letters denote significant differences (a/b/c: different treatments of passive pigs; A/B/C: different treatments of active pigs)

### Change in the heart rate of pigs

Table 5 shows the effect of different treatments of active pig and passive pig on the change in the heart rate of pigs. Different treatments of active pig have an impact on the change in the heart rate of active pig. The reward treatment results in the highest average of change in the heart rate compared to the other treatments (*p* < 0.01). Within passive pig reward group, the reward treatment results in the highest change in the heart rate compared to the other treatments (*p* < 0.01). Within passive pig punishment group, the punishment treatment results in the lowest change in the heart rate compared to the other treatments of active pig (*p* < 0.01). Within passive pig no treatment group, no treatment results in the lowest change in the heart rate compared to the other treatments of active pig (*p* < 0.01). Different treatments of passive pig have an impact on the change in heart rate of active pig. All treatments differ significantly from each other on the change in the heart rate, with punishment treatment causing the lowest change in the heart rate and no treatment the highest (*p* < 0.01). Within active pig reward group, the punishment treatment of passive pig results in the lowest change in the heart rate compared to the other treatments (*p* < 0.01). Within active pig punishment group, no treatment results in the highest change in the heart rate compared to the other treatments (*p* < 0.01). Within active pig no treatment, no significant difference between treatments (*p* > 0.05).

**Table 5:** Change in the heart rate of pigs (*n* = 4)

		PRT	PPT	PNT	Average	*p*
Active Pig	ART	14.85 ± 2.01^Aa^	2.08 ± 1.55^Ab^	15.61 ± 1.99^Aa^	10.85 ± 1.19^A^	< 0.01*
	APT	-3.20 ± 1.41^Bb^	-4.58 ± 1.10^Bb^	11.76 ± 2.84^Aa^	1.33 ± 1.27^B^	< 0.01*
	ANT	-2.27 ± 1.83^B^	2.26 ± 1.76^A^	-2.18 ± 1.55^B^	-0.73 ± 1.00^B^	0.11
	Average	3.13 ± 1.23^b^	-0.08 ± 0.90^c^	8.40 ± 1.41^a^		< 0.01*
	*p*	< 0.01*	< 0.01*	< 0.01*	< 0.01*	
Passive Pig	ART	13.22 ± 2.61^Aa^	0.71 ± 1.53^Ab^	-5.45 ± 1.19^c^	2.83 ± 1.26^A^	< 0.01*
	APT	-4.65 ± 2.01^C^	-1.45 ± 1.92^AB^	-6.64 ± 1.20	-4.25 ± 1.02^C^	0.11
	ANT	5.31 ± 3.00^Ba^	-4.92 ± 1.46^Bb^	-3.24 ± 1.42^b^	-0.95 ± 1.26^B^	< 0.01*
	Average	4.62 ± 1.60^a^	-1.88 ± 0.97^b^	-5.11 ± 0.74^c^		< 0.01*
	*p*	< 0.01*	0.05*	0.18	< 0.01*	

*ART* Reward treatment of active pig, *APT* Punishment treatment of active pig, *ANT* No treatment of active pig, *PRT* Reward treatment of passive pig, *PPT* Punishment treatment of passive pig, *PNT* No treatment of passive pig. Mean ± standard error. Different letters denote significant differences (a/b/c: different treatments of passive pigs; A/B/C: different treatments of active pigs)

Different treatments of active pig have an impact on the change in the heart rate of passive pig. All treatments differ significantly from each other on the average change in the heart rate, with the punishment treatment causing the lowest average change in the heart rate and the reward treatment the highest (*p* < 0.01). Within passive pig reward group, all treatments differ significantly from each other on the change in the heart rate, with the punishment treatment causing the lowest change in the heart rate and the reward treatment the highest (*p* < 0.01). Within passive pig punishment group, the reward treatment results the change in heart rate significantly greater than in no treatment (*p* = 0.05). Different treatments of passive pig have an impact on the change in the heart rate of passive pig. All treatments differ significantly from each other on the average change in the heart rate, with no treatment causing the lowest average change in the heart rate and the reward treatment the highest (*p* < 0.01). Within active pig reward group, all treatments differ significantly from each other on the average change in the heart rate, with no treatment causing the lowest average change in the heart rate and reward treatment the highest (*p* < 0.01). Within active pig no treatment group, the reward treatment results in the highest change in the heart rate compared to the other treatments (*p* < 0.01). There were no significant differences between the different treatments in the remaining groups (*p* > 0.05).

**Fig. 3:**
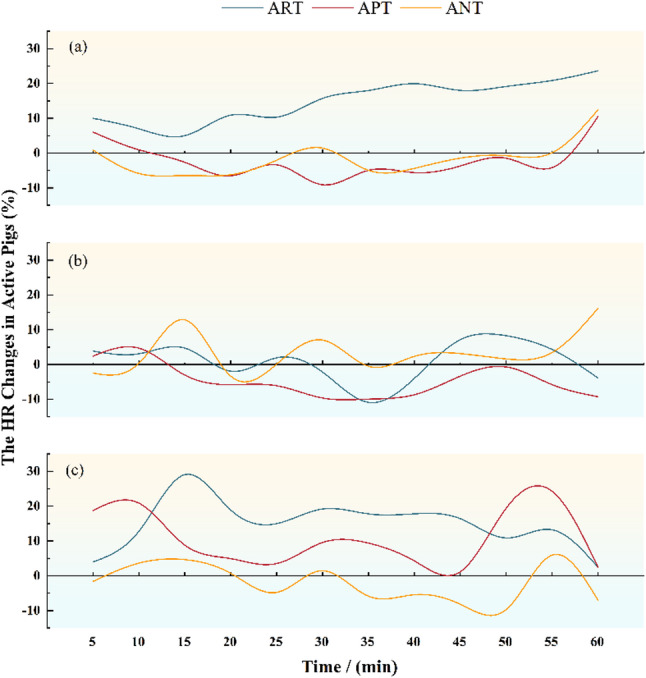
Change in the heart rate of active pigs (*n* = 4). **a** passive pig reward group; (**b**) passive pig punishment group; (**c**) passive pig no treatment group. ART: Reward treatment of active pig; APT: Punishment treatment of active pig; ANT: No treatment of active pig

As shown in the Fig. 3, within passive pig no treatment groups, different treatments of active pig results in generally similar change curves in the heart rate, with smoother heart rates in active pig no treatment. Both reward and punishment treatments significantly increased heart rate in pigs. The increase is greater in the reward treatment of pigs, while faster in the early part, it gradually levelled off in the later part; the change in increase was more pronounced in the punishment treatment of pigs. Within passive pig punishment groups, the heart rate changes significantly in active pig no treatment. The punishment treatment results in lower heart rates in piglets. The reward treatment results in a greater change in the heart rate, but the overall heart rate was smoother in the piglets. Within passive pig reward groups, the active pig reward treatment results in gradually increasing in the heart rate; the change in the heart rate of pigs under the influence of the punishment treatment and no treatment is smoother, but the heart rate decreases in general.

**Fig. 4:**
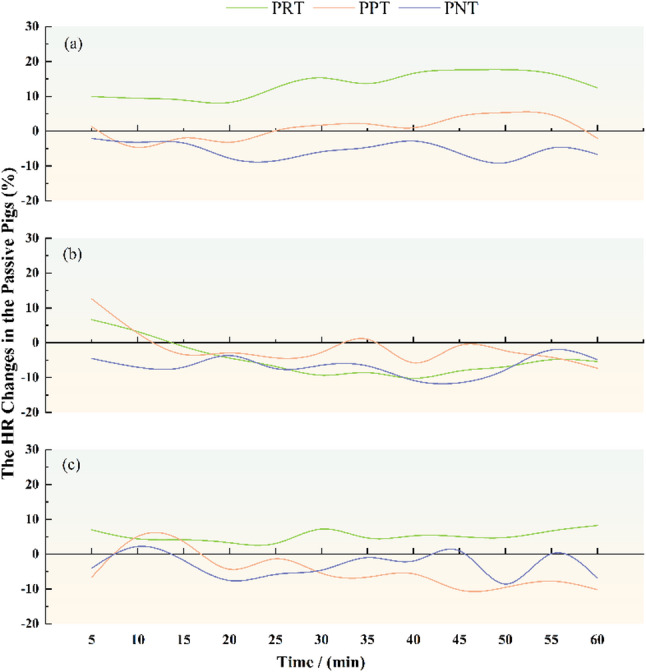
Change in the heart rate of passive pigs (*n* = 4). **a** active pig reward group; (**b**) active pig punishment group; (**c**) active pig no treatment group. PRT: Reward treatment of passive pig; PPT: Punishment treatment of passive pig; PNT: No treatment of passive pig

As shown in the Fig. 4, within active pig reward groups, different treatments of passive pig resulted in broadly similar changes in the heart rate, with the reward treatment resulting in an overall increase in the heart rate, the punishment treatment resulting in a smoother heart rate, and an overall decrease in the heart rate under no treatment. Within active pig punishment groups, the heart rate decreased overall in all cases, but both the reward treatment and the punishment treatment led to an increase in the heart rate followed by a decrease in the heart rate. Within active pig no treatment groups, both the punishment treatment and no treatment resulted in a decrease in overall heart rate, while the reward treatment resulted in an increase in the heart rate.

## Discussion

The present study aimed to investigate whether piglets exhibit altruistic behaviour that may be underpinned by cognitive empathy. Specifically, we examined whether the behaviour of the active pig was influenced by the treatment received by the passive pig, and whether the behavioural and physiological responses were consistent with an empathy-related process. Overall, the findings suggest that the active pig adjusted its button-pressing behaviour according to the treatment experienced by its peer, as reflected in the significant interaction between active pig treatment and passive pig treatment. Notably, when the passive pig was subjected to punishment, the active pig reduced its button pressing, even when pressing the button resulted in a reward for itself. These results support the possibility that piglets may regulate their own behaviour in response to the emotional state of a conspecific.

### Differences in pig motivation to explore and frequency of button presses

The button-pressing behaviour was observed in all active pigs involved in the experiment. Curiosity is a psychological response to uncertainty [21]. All the piglets showed curiosity about the buttons in the test environment, perhaps because the buttons did not exist in the environment when the piglets were familiarized with the test environment. Exposure to unfamiliar stimuli can elicit exploratory responses in piglets, as they demonstrate attentiveness and increased interaction with novel objects in standard novelty‑preference paradigms, reflecting an intrinsic motivation to investigate the unfamiliar rather than mere habituated behaviour [22, 23]. The button constituted a novel stimulus for the piglets during the test, which stimulated the curiosity of the piglets and made them show exploratory behaviour for pressing the button. This indicates that the active pig frequently approached and explored the button, reflecting curiosity towards the novel object. When passive pigs were not treated, the distinct treatments of the active pig yielded a notable discrepancy in the frequency of button presses. This is supported by the observation that active pigs subjected to the punishment treatment pressed the button markedly fewer times than those in the reward and no-treatment groups. This difference may be due to the fact that low-intensity negative stimuli trigger the animal’s curiosity which leads to exploratory behaviour [24], whereas high-intensity negative stimuli lead to fear [25]. Additionally, some scholars have placed curiosity in a contrast with anxiety, arguing that the former involves approaching new things out of interest and is a counterweight to the latter, which involves avoiding new things out of fear [26]. Cognitive processes can be regarded as ‘components’ of human emotion states or events, both in the form of appraisals which can trigger the occurrence of particular emotions, and cognitive outputs which can result from emotional states [27]. Different treatments delivered after button pressing may have shaped the emotional valence associated with the action, thereby influencing the likelihood of subsequent button presses.

### Emotional arousing in piglets after pressing the button

After the active pig pressed the button, the differently treated active pig and passive pig were exposed to different treatments, resulting in corresponding behaviours and changes in heart rate. Behavioural measures of emotional state in pigs, which can provide a better indication of the valence of experienced emotions [28]. Freezing and escape attempts are commonly interpreted as behavioural indicators of fear in pigs [14]. The administration of punishment treatment to piglets resulted in a notable elevation in both the incidence of freezing and the frequency of escape attempts. This finding suggests that the application of such stimuli may have elicited a negative emotional response, potentially comprising fear, distress, or alertness, in the subjects, thereby contributing to an increase in the incidence of freezing. Piglets possess an innate drive to seek avoid harm. Piglets exhibit behavioural inhibition and reduced exploratory activity when exposed to novel or elevated environments, which are generally interpreted as adaptive defensive responses to perceived threat [29]. When pigs perceive that their immediate environment is not a secure one [14, 30], they will demonstrate a greater propensity to escape attempts to flee. One study found that fear may trigger rigidity in animals thus causing their heart rate to slow down [31]. In our study, both active pig and passive pig subjected to the punishment treatment showed a significant decrease in heart rate. Boissy et al. suggest that cardiac vagal tone is a potential indicator of positive emotion and thus of the affective valence dimension [32]. The vagus nerve plays an important role in the autonomic regulation of cardiac activity and has been associated with emotional processing in animals, particularly through its contribution to sympathovagal balance [33, 34]. The results of the experiments demonstrated that both active pig and passive pig stimulated by rewards exhibited varying degrees of heart rate increase, with the increase being significantly higher than that observed in the punishment and no treatment piglets. These results indicate that food rewards effectively elicited emotional arousal in the piglets.

The observed differences in heart rate changes may partly reflect emotional contagion between piglets. Heart rate is widely recognised as an indicator of autonomic regulation and emotional arousal [34]. When one individual experience emotional activation, its affective state may influence its conspecific, thereby modulating physiological responses. Such mutual reinforcement could amplify existing arousal, consistent with findings in humans showing additive emotional effects in social contexts [35]. However, the magnitude of heart rate changes suggests that, although emotional contagion was present, the direct impact of the external stimulus on emotional arousal was more substantial.

### Emotional contagion and social support in pigs

The results showed that pigs subjected to the punishment treatment displayed more freezing and escape attempts, suggesting that the aversive stimulus induced a negative affective state, potentially associated with pain, fear, or anxiety. Such an emotionally negative affective state may have motivated the piglets to seek proximity to their peers as a coping strategy [13]. Previous studies have indicated that pigs tend to increase social contact when exposed to challenging or aversive situations [13, 36]. Nose-to-nose contact is generally regarded as a form of affiliative social behaviour requiring close physical proximity between the active and passive pigs. In the present study, punishment treatment of pigs exhibited a higher frequency of nose-to-nose contact, which may suggest an increased motivation to engage in social interaction under negative conditions, possibly reflecting a form of social buffering.

In the active pig punishment group, active pigs who witnessed their peers being punished exhibited more freezing, indicating that the fear of their peers can intensify the piglets’ own fear, thereby demonstrating the phenomenon of emotional contagion between pigs. The emotional contagion was not exclusive to interactions of negative emotion; in the case of active pig punishment group, the impact of passive pig treatment on active pig’s heart rate variability and physiological level were observed. The changes in the active pig’s heart rate were significantly lower when the passive pig received treatment, irrespective of the type of treatment administered to the passive pig. At the same time, the freezing of the active pig was significantly reduced when the peer received a reward treatment or no treatment. When the passive pig received reward treatment, punishment treatment of active pigs approached the passive pig less frequently than both reward treatment and no treatment of active pigs. This difference may be associated with the negative emotional state induced by the punishment treatment in active pig punishment treatment, which could have led to reduced activity. Furthermore, irrespective of the treatment received by the active pig, the change in heart rate was significantly lower when confronted with a peer that received the punishment treatment compared to when confronted with a peer that received no treatment. Both the active pig and the passive pig showed a significant increase in nose-to-nose contact behaviour in pigs subjected to punishment treatments, suggesting that while piglets actively seek peer social support, peers are also able to proactively provide social support as a way to help alleviate fear. Similar to our findings, Söderquist et al. found in pigs that the presence of a peer appeared to attenuate the response of subject piglets to an aversive stimulus (bubbles), as evidenced by the appearance of lower levels of activity and fewer escape attempts as well as more time spent in close proximity to the peer, suggesting that the presence of a peer does indeed provide social support to a distressed peer, thereby alleviating the distressed peer’s emotional state [37]. This is consistent with the findings of Langford et al. on laboratory mice that the presence of a peer may have an analgesic, palliative effect on mice in pain [38]. These studies all suggest that social support from healthy peer can mitigate the effects of stress on animals, and therefore this may be a key method of promoting positive physical and psychological well-being in animals.

### Cognitive and altruistic behaviour in pigs

Paul et al. define cognition as “the mechanisms by which animals acquire, process, store and act on information from the environment” [27]. Cognitive processes can be regarded as ‘components’ of human emotion states or events, both in the form of appraisals which can trigger the occurrence of particular emotions, and cognitive outputs which can result from emotional states [27]. In our experiments, when the passive pig was untreated, different treatments of the active pig resulted in significant differences in the frequency of button presses for the active pig. In the previous section, we have shown that pigs can associate rewards or punishments with button presses, which also reflects their cognitive abilities. Because of individual distress is more often associated with self-directed behaviour (escaping a distressing situation to terminate one’s own distress), individual distress causes individuals to become more self-conscious, which affects individual behaviour [39]. Therefore, when the active pig is subjected to punitive treatment, its behaviour of stopping pressing the button may also be an attempt to reduce his own suffering, i.e. a manifestation of ‘self-interested behaviour’.

It has been suggested that altruistic behaviour in animals is driven by a desire for social contact [1, 40]. However, our results found that when the passive pig was subjected to punishment treatment, the number of times the active pig approached the barrier was significantly reduced and the freezing was significantly increased compared with the passive pig with no treatment, suggesting that the active pig was not willing or afraid to approach the passive pig at this time. Therefore, we believe that the active pig stopped the button press not to interact with the passive pig, but because he felt the passive pig’s negative emotions such as fear and pain. At this point the active pig may have been affected by the passive pig’s negative emotions and developed negative emotions and was in a state of some degree of social avoidance. However, when active pigs perceived that their peers were experiencing punishment treatment, they displayed pro-social behaviour, i.e., they ended their peers’ distress by stopping the button press. Regardless of what treatment the active pig received after the button press, the button pressed by the active pig was significantly lower when the passive pig received the punishment treatment than in the passive pig reward and no treatment groups. This would seem to demonstrate that no matter what the active pig itself experiences, when confronted with the suffering of its peers, the active pig is able to link the conditions that trigger the suffering of its peers to its own button press, which in turn makes the suffering of its peers less painful by reducing the frequency it presses the buttons. In a study of altruistic behaviour in rodents, Cox et al. used a model that could remove the effects of social interactions and demonstrated that altruistic behaviour in rats is driven by empathy and would help a distressed peer even in the absence of social rewards [41]. In our experiments, there was an effect of the different treatments experienced by the passive pig on the frequency of button presses of the active pig when the active pig received a reward treatment. When the passive pig was subjected to the punishment treatment, the active pig’s button presses were highly significantly lower than those of the passive pig reward and no treatment groups. In this case, since the active pig was rewarded with food when it pressed the button, we hypothesized that the difference occurred because the active pig found that its button press caused its peer to suffer from the pain of the electric shock, and therefore reduced the frequency of button presses even though stopping to press the buttons prevented the active pig from obtaining food itself. This caused piglets to voluntarily stop the behaviour when they sensed that they were causing pain to their peer as a result of their behaviour. Behaviourally it is stop to press button at this point that is indeed an altruistic behaviour, which is similar to the findings of Cox et al. [41]. The active pig stops the button press perhaps because it senses the peer’s distressing emotion and understands the source of the peer’s distressing emotion and stops the button press to prevent the peer from distressing again, and so it can be inferred that the active pig stops the button press at this point in time as an altruistic behaviour triggered by an empathetic response. However, it remains to be determined another layer of the reason for the active pig’s stop button press may be that the negative emotions of the peer due to the punishment treatment brought emotional discomfort to the active pig itself, because at this time the active pig freezing increased significantly, suggesting that the active pig may also be in the midst of negative emotions at this time; stopping the button press may be a behaviour that the active pig tries to minimize its own pain, i.e., a manifestation of altruistic behaviours driven by self-interest, but further studies are required by follow-up studies.

## Conclusion

In conclusion, the experiments demonstrated that pigs could associate the emotional states of their peers with their own behaviours. This indicated that they were able to discern the source of their peers’ emotions. Furthermore, when they identified that their own behaviour could evoke negative emotions from their peers, the pigs could opt to terminate their behaviours to end their peers’ suffering caused by their own actions. This is an exemplification of the existence of altruistic behaviours based on cognitive empathy in pigs. This suggests that improving pig welfare requires attention to their social and emotional needs, and that group dynamics need to be emphasised in production to reduce the negative effects of stress through social support between peers in the group.

## Data Availability

Upon reasonable request, the datasets of this study can be available from the corresponding author.

## Abbreviations

ART: Reward treatment of active pig
APT: Punishment treatment of active pig
ANT: No treatment of active pig
PRT: Reward treatment of passive pig
PPT: Punishment treatment of passive pig
PNT: No treatment of passive pig
